# Perinatal risk and the cultural ecology of health in Bihar, India

**DOI:** 10.1098/rstb.2019.0433

**Published:** 2020-06-29

**Authors:** Cristine H. Legare, Santosh Akhauri, Indrajit Chaudhuri, Faiz A. Hashmi, Tracy Johnson, Emily E. Little, Hannah G. Lunkenheimer, Alexandra Mandelbaum, Harsh Mandlik, Sudipta Mondal, Nachiket Mor, Neela Saldanha, Janine Schooley, Priyam Sharda, Shalini Subbiah, Siddharta Swarup, Mari Tikkanen, Oskar Burger

**Affiliations:** 1 Department of Psychology, The University of Texas at Austin, Austin, TX, USA; 2 Project Concern International, San Diego, CA, USA; 3 Bill and Melinda Gates Foundation, Seattle, WA, USA; 4 Scope, Helsinki, Finland; 5 Banyan Academy of Leadership in Mental Health at Kanchipuram, Tamil Nadu, India; 6 Centre for Social and Behaviour Change, Ashoka University, Sonipat, Haryana, India

**Keywords:** folkbiology, India, maternal-child health, perinatal health, ritual, traditional medicine

## Abstract

The objective of the current study is to examine the cultural ecology of health associated with mitigating perinatal risk in Bihar, India. We describe the occurrences, objectives and explanations of health-related beliefs and behaviours during pregnancy and postpartum using focus group discussions with younger and older mothers. First, we document perceived physical and supernatural threats and the constellation of traditional and biomedical practises including taboos, superstitions and rituals used to mitigate them. Second, we describe the extent to which these practises are explained as risk-preventing versus health-promoting behaviour. Third, we discuss the extent to which these practises are consistent, inconsistent or unrelated to biomedical health practises and describe the extent to which traditional and biomedical health practises compete, conflict and coexist. Finally, we conclude with a discussion of the relationships between traditional and biomedical practises in the context of the cultural ecology of health and reflect on how a comprehensive understanding of perinatal health practises can improve the efficacy of health interventions and improve outcomes.

This article is part of the theme issue ‘Ritual renaissance: new insights into the most human of behaviours’.

## 1. Introduction

The perinatal period is associated with substantial health risks to mothers and infants. These risks vary in amount and kind based on environmental risk factors and access to high-quality healthcare. Maternal and infant mortality rates have declined rapidly over the past several decades worldwide, but continue to vary substantially among populations [1]. For example, in 2017, the maternal mortality ratio (maternal deaths per 100 000 live births) was 917 in Nigeria, 145 in India and 3 in Finland [1]. In 2017, the infant mortality rate (per 1000 live births) was 77 in Nigeria, 32 in India, and 3 in Finland [2–4]. Maternal and infant mortality rates also vary substantially within populations. Despite a commendable 77% nationwide decline in maternal and infant mortality rates in India in the past two decades [1,5], in the predominately rural state of Bihar, the maternal mortality ratio was 165, and the infant mortality ratio was 35 in 2017 [5].

High levels of risk require strategies for reducing it and alleviating the anxiety associated with perceived threats. All human populations use a diverse repertoire of traditional and biomedical health practises in response to risk or to mitigate negative outcomes [6,7]. Research on folkbiological concepts [8] has shown that introducing biomedicine into populations does not replace traditional (folk) medicine or supernatural explanations for health [7,9–13]. Instead, traditional medicine and biomedicine *coexist.* For example, although information about the transmission of the acquired immunodeficiency syndrome (AIDS) virus is widely available via health and education programmes in South Africa [14], supernatural accounts of infection based on witchcraft are commonplace [7,15,16]. Traditional medicine and biomedicine are often used to explain outcomes or events at different levels of causality. In the context of AIDS in South Africa, supernatural explanations such as witchcraft provide distal explanations of why human immunodeficiency virus (HIV) was contracted; biomedical explanations such as germ theory provide proximate explanation for how it was contracted, for example, ‘a witch can make a condom weak, and break’ [7].

An especially informative domain for studying the coexistence of natural (biomedical) and supernatural practises is perinatal health. Most disease is caused by multiple factors [17]; biological, social, supernatural and religious explanations are used for illness transmission [18–20]. Contagion can also occur through physical, supernatural, mental or moral processes. Illness often has a human vector and thus can be both an interpersonal and a biological process [21]. For example, witchcraft and biological contagion are widely used by the same individuals to explain the same instances of illness [16,22].

Globalization is changing the cultural ecology of perinatal health throughout the world, resulting in the introduction of biomedical treatments for illness and infirmity into populations with rich and complex traditional health ecologies and perinatal health practises, many of which are infused with religious meaning. In some cases, biomedical health practises supplant traditional perinatal medical practises. In other cases, biomedical health practises and traditional perinatal medical practises coexist in ways that range from conflict to complementarity [11,13]. For example, in a recent 11 country study of habitual health-promoting behaviour (e.g. hand-washing with soap), one of the barriers to the adoption of a biomedical practise was a religious ritual (e.g. hand-washing with plain water as part of ritual cleansing) [23].

In Bihar, India, multiple kinds of healthcare are available including traditional medicine, religious healing and biomedical services. Perhaps the most prominent supernatural explanation for misfortune in this context is evil eye, *ku drishti* or *buri nazar*. Evil eye is a curse caused by a malicious gaze, usually when a person is unaware, and is believed to cause a wide variety of misfortunes, including illness and death [24]. Charm bracelets, amulets, markings on the face or body, or slogans are used to ward off the evil eye.

Our objective is to document and describe the complex, diverse and wide-ranging health ecology associated with mitigating risk during the perinatal period. There are traditional perinatal medical practises in all human populations, but we propose that the amount and kind of beliefs and behaviours are influenced by local ecologies of health and the amount and kinds of local risk [25–27]. The goals of perinatal health practises in high-risk contexts are often protective or promotive: to avoid negative outcomes, such as miscarriage, difficult labour, or birth defects, or to achieve positive outcomes, such as infant and maternal wellbeing and health. We document health-related beliefs and behaviours in a population experiencing substantial risk to maternal and infant health, and rapid changes in healthcare. Bihar is India's most rural state, with over 88% of the population living in rural settings. It is also India's poorest state, ranking 33rd out of 33 in gross state domestic product per person. Bihar has a high population density by global standards with over 1100 people per square mile and is one of India's fastest growing states, with a decadal growth rate of just over 25% [28].

Traditional and biomedical healthcare systems exist in parallel in Bihar. Biomedicine is now widely practised, and has been incorporated into the repertoire of traditional perinatal health practises used in this region. Community members frequently use a range of services from different sectors. For example, it is now common for women to give birth in medical clinics (biomedical healthcare system) [1] and call the Dai (traditional birth attendant) at home to confirm that their labour has begun prior to going to the hospital. In recognition that India's rural states are medically underserviced and experiencing poorer health outcomes, the government of India launched the National Rural Health Mission (NRHM) in 2005. The NRHM introduced several initiatives aimed at improving the health outcomes of rural Indian populations, including employing health activists and practitioners to provide health education and services, such as vaccination, nutritional supplements, prenatal vitamins, institutional delivery, support for immediate and exclusive breastfeeding, and recommendations for infant caretaking practises to women of reproductive age [29].

Practises associated with traditional medicine, such as perinatal ritual, may be the byproduct of a *hazard precaution system*, that functions to respond to inferred threats, including social (e.g. ostracism) and physical (e.g. contamination and pathogens) dangers [30]. We hypothesize that the perceived function of these practises (by practitioners) in high-risk contexts is primarily to avoid danger rather than promote health. Traditional health practises include taboos, superstitions, religious and folk-medical practises, and rituals; all of which operate as culturally sanctioned responses to perceived threat [30,31]. Notably, these traditional medical practises share many of the same characteristics. For example, they are socially transmitted, they operate based on principles of magical contagion and are often opaque from the perspective of physical causality [7,32,33]. Like traditional medicine, biomedical practises are also perceived to be causally opaque by most of the lay population (in rural northern India and everywhere in the world), are socially transmitted, and are heavily ritualized. For these reasons, we propose that biomedicine is a new kind of ritual repertoire that coexists with traditional medical practises.

Rituals, which we define as socially stipulated, normative behaviour, are of particular interest in the context of health risk mitigation during the perinatal period. They are widely practised for protective, restorative and instrumental or goal-directed purposes around the world [34–36]. Evidence for the use of rituals to treat health-related problems dates back to at least ancient Egypt [37]. Rituals continue to be practised by contemporary populations to treat health problems as diverse in etiology as AIDS, tetanus and tuberculosis [6,7,32,36]. Using rituals for instrumental purposes, such as mitigating threat, requires entertaining their potential for causal potency or efficacy [35,38,39]. Rituals often involve attention to perceived danger and prescriptive, rigid behavioural patterns for averting threat [40]. Pregnancy and birth are both fitness-relevant events; thus, the perinatal period provides unique insight into causal and functional relations of traditional medical practises such as rituals relevant to maternal and child health.

Perinatal rituals serve multiple functions within communities [41,42]. Consider Chhathi, a perinatal ritual widely practised throughout Bihar, India, on the 6th day after birth. It serves social functions; it marks critical transitions in the lifecourse (birth and parenthood), it marks the initiation of the infant into the family, it has symbolic meaning to ritual practitioners and it reinforces social cohesion within the community [43]. It also has instrumental functions; it is believed to reduce the risk of negative outcomes for the new baby (e.g. ward off the threat of evil eye).

In the current study, we document the occurrence, objectives and explanations of health-related beliefs and behaviours during pregnancy and postpartum using focus group discussions with younger mothers and older mothers in Bihar. First, we document perceived physical and supernatural threats and beliefs about how traditional and biomedical practises mitigate them. Second, we describe the extent to which these practises are explained in the context of risk-preventing versus health-promoting behaviour. Third, we discuss the extent to which traditional perinatal medical practises are consistent, inconsistent or unrelated to biomedical health practises and describe the extent to which traditional and biomedical health practises compete, conflict and coexist. Finally, we conclude with a discussion of the relationships between traditional and biomedical practises in the context of the cultural ecology of health and reflect on how a comprehensive understanding of perinatal health practises can improve the efficacy of health interventions and improve outcomes.

## 2. Methods

The data presented below are part of a larger research initiative called Project RISE, which aims to harness Ritual to Improve Service-delivery and Empower health workers in Bihar, India. Here, we report data from focus group discussions that are the basis for our description of health-related beliefs and behaviours. The goal of the focus group discussion data reported in this paper was to gather rich qualitative information about perceived risks and health-related practises during the perinatal period.

### (a) Participants

The Indian state of Bihar has 38 districts which are divided into 534 administrative blocks. Most blocks contain several villages. A community development block covers several *gram panchayats,* the local administrative unit at the village level. Participants were recruited from 21 villages in three blocks from two districts. Participants from four villages in the blocks of Biharsharif in the Nalanda district, six from the Rajgir Block of Nalanda District and 11 from the Warisnagar Block of Samastipur District participated in the focus group discussions. These villages were randomly selected from two linguistic sub-divisions. Forty focus group discussions were conducted in total, 20 with younger mothers and 20 with older mothers. For the younger mother sample, women had to have given birth within the last 2 years. The older mother sample is comprised mothers who have a married daughter or daughter-in-law who has given birth within the previous 2 years. Attempts were made to ensure that a locally representative sample of age, religion, parity and caste was obtained for each sample. The focus group discussions ranged in size from four to seven participants with a median of five, in addition to the trained researcher and a note-taker. In total, Project Concern International (PCI) investigators interviewed 107 younger mothers (mean age: 24, range: 18 to 35) and 106 older mothers (mean age: 52, range: 35 to 75). The younger mother focus group discussions were composed of 69 Hindu and 38 Muslim women. The older mother focus group discussions had 68 Hindu and 37 Muslim women. Most of the focus group discussions had exclusively Hindu or Muslim participants in order to compare the differences and similarities between perceptions of health behaviours between religious groups.

### (b) Procedure

The focus group discussions were conducted by experienced rural health researchers employed by PCI, an international non-government organization with extensive experience working in Bihar. Prior to beginning data collection, PCI gave a week-long extensive training to the data collection team on the focus group discussion tools and protocol. Conversations were audio recorded for quality assurance and transcriptions. Basic demographic information was recorded on all participants. Researchers used a standardized set of checklists and followed uniform guidelines to ensure a consistent dialogue across focus group discussions, and used follow-up questions to resolve ambiguity and ensure the discourse was free-flowing. The researchers collected data during a three week period in January, 2019.

Participants were asked to discuss health-related beliefs and practises, to describe when in the perinatal period (pregnancy and early postpartum) they occurred, and to explain why they are practised. Beliefs about common threats to maternal and child health and health-related practises were discussed, as were traditional perinatal rituals and biomedical practises. Preferences for public, private and home-based care were addressed at each stage of the perinatal period. Participants were encouraged to describe health-related beliefs in the context of beliefs about health outcomes they promote or risks they mitigate. Both home and community rituals were discussed.

### (c) Coding

The focus group discussions were fully transcribed from audio-recordings to Hindi, then excerpts were translated to English by researchers fluent in Bihari dialects of Hindi and English. In total, data processing resulted in 720 individual descriptions that were consolidated to identify 269 distinct health-related practises. Note that utterances from these data were recorded in a group context and then coded; thus, we are not reporting verbatim quotes that are assignable to individual participants. Rather, the data are a mixture of quotes and paraphrases of utterances made during focus group discussions. The procedure for analysing the data included coding, sorting, counting and describing the results of targeted queries. For instance, each utterance is linked to its respective focus group discussion and point in the perinatal period.

Conversational content was coded for descriptions of physical and supernatural threats to maternal and child health, as well as traditional and biomedical practises. The following codes were applied to the perinatal practises: common threats to physical health, common practises believed to mitigate physical and supernatural threat, including whether they involved placing a visible marker on the body or home as a social signal or for supernatural protection, and the birth ritual of Chhathi. Data were coded for whether the health-related practises were risk avoiding or health promoting. Risk-avoiding practises are intended to avoid or prevent a harmful outcome. Health-promoting practises are intended to improve visible, physical and psychological health. Note that a small number of practises that were not explicitly health promoting or risk avoiding (e.g. practises believed to influence the gender of the infant) were coded as unrelated and not included in table 1. Data were also coded for the extent to which practises are consistent, inconsistent or unrelated/neutral with current biomedical recommendations. Note that practises related to food recommendations and taboos were not included in this dataset and are not included in the relationship to recommended biomedical practises in table 1. Examples of practises relevant to maternal and child health that involved replacement of traditional medical practises with biomedical recommendations, conflict between traditional medical and biomedical recommendations, and coexistence of traditional medical and biomedical recommendations were also documented.

**Table 1.** Counts of health-related practises, descriptions as health promoting or risk avoiding, and relationship to recommended biomedical practises.

descriptor	pregnancy		postpartum	
	younger mothers	older mothers	younger mothers	older mothers
count of practises	155	206	151	210
health promoting	19 (12%)	29 (14%)	67 (44%)	103 (49%)
risk averting	136 (88%)	177 (86%)	84 (56%)	101 (48%)
neutral re biomedical	104 (67%)	150 (73%)	91 (60%)	120 (57%)
consistent with biomedical	47 (30%)	49 (24%)	20 (13%)	31 (15%)
contra biomedical	1 (1%)	7 (3%)	36 (24%)	45 (21%)

## 3. Results

Nearly every belief and practise discussed during the focus group discussions described a health-related outcome or concern (table 1). These health-related practises varied considerably and ranged from traditional perinatal rituals based on local customs, to taboos and superstitious practises believed to ward off physical and supernatural threat, to cultural beliefs about impurity of mother and child after birth, and to practises based on biomedical recommendations.

### (a) Traditional medical practises believed to mitigate physical and supernatural threats to mother and child

Hindu and Muslim mothers reported wide-ranging taboos and superstitions believed to mitigate physical threats to mother and child. Reasons reported for these practises were consistent across regions and groups. Miscarriage, birth complications and premature delivery were frequently mentioned concerns for both younger and older mothers during the pregnancy period. Taboos and superstitious practises with the intent of mitigating these risks included avoiding lifting heavy objects, labour intensive housework, climbing stairs, sexual activity and travelling out of the home. Night time may be an especially dangerous time for women to leave the house and a commonly stated reason for pregnant women to avoid doing so was risk to mother and child associated with the encountering the supernatural *jiloi bird.* Participants described the jiloi bird as ugly and dangerous, and distinguished it from bats and owls. Mothers reported that pregnant women should avoid the risk of being outside when the bird flies overhead at night or in the early morning for fear that exposure to the bird may cause the child to be weak, thin or born with a foul odour. If a pregnant woman is exposed to the jiloi bird during pregnancy, there are remedies one can do postpartum to rid the baby of the negative consequences. For example, bathing an infant in a sugarcane field and replacing their old clothes, or placing dried dung of equivalent weight to the infant in a river.

Postpartum women and infants are considered impure, thus a number of taboos are practised to prevent contaminating others. For example, new mothers refrain from worship and avoid touching water or food sources because of their impure status. Places like temples, handpumps and kitchens are considered pious and pure, thus postpartum women should avoid them.

There are many taboos associated with astrological beliefs about dangers to pregnant women and their fetuses during solar and lunar eclipses. For example, several mothers stated pregnant woman should avoid cutting things during an eclipse. Stated reasons were consistent with associational magic, such as risks that newborns will have cuts on their bodies or other physical deformities. Several mothers also reported that pregnant women should not eat or sleep during an eclipse to avoid their children developing physical deformities. Placing amulets in the home during an eclipse is commonplace. For example, mothers reported that one should hang a piece of thread or wood equivalent to the height of the pregnant woman on the wall during an eclipse.

Hindu and Muslim mothers frequently reported taboos and superstitions associated with avoiding evil eye during pregnancy and the postpartum period. Evil eye is a supernatural curse that is believed to be transmitted through a malicious gaze and is usually motivated by envy, jealousy or lack of reciprocity that may cause illness or even death [7,44]. Applying visible, physical amulets and substances to the body or the home of the infant and mother to ward off evil eye is commonplace. Taboos for pregnant women to avoid to reduce the risk of evil eye included not walking outside, not looking at or crossing rivers and not reporting pregnancy to others. Many considered rivers to be open or barren which are believed to be more prone to threats from spirits or demons. Another common risk-avoiding practise reported by mothers is tying a locket around the neck or arm of the mother and infant. During the birthing period, mothers reported that bringing an iron item to the hospital, such as a knife, would protect their newborns from evil eye. Upon returning to their homes post-delivery, younger and older mothers reported burning wood at the entrance of the family home and keeping iron objects outside of the room the mother and infant sleep in. Adornments applied to the body like asafoetida (‘devil's dung', dried gummy excretion from the tap root of ferula), and tying black thread to the arm of mother and child were frequently discussed.

### (b) Birth ritual: Chhathi

Chhathi is a ritual widely practised in northern India to initiate a new baby into the family. Appeals to supernatural agents for protection and blessings are central to the Chhathi ceremony for Hindu and Muslims mothers. Hindus believe in reincarnation, thus some of the practises associated with Chhathi relate to pardoning the sins committed during previous lives, and beginning a new life by giving the child a name and consulting horoscopes to decide their fate in this life. Muslims do not believe in reincarnation, thus some of the elements of Chhathi are instead associated with transitioning the infant from God's world to the world of the living. Mothers also reported that the newborn should touch a pen during Chhathi. Hindu mothers indicated that this was done so that the God of Creation will record an auspicious future for the infant. Muslim mothers performing this ritual believe that Allah should be invited to decide the fate of the child. Prior to this celebration, both mother and child are bathed. While the mother receives new clothing, the child is wrapped in a cloth and rolled on the floor for a short period of time. Hindu mothers reported that the village *pandit* gives the child their horoscope. New mothers receive special and diverse foods during Chhathi. Five to seven types of vegetables, pulses (legumes), rice and fish are prepared for the mother to eat. The child is kept in the mother's lap so that she is able to touch her lips to the child's mouth after she has eaten so the baby tastes the food. Doing this is meant to ensure that the child will never remain hungry in life. Black thread is to be worn by the baby around the waist or wrist to help strengthen and increase the lifespan of the child. Kohl is applied around the eyes of the baby to increase beauty, intelligence, and to protect the child from evil eye.

### (c) Risk aversion versus health promotion

Nearly every health-related practise could be described as having the goal of promoting the health of the mother or child or of averting risks that could negatively impact the health of mother or child. Risk-avoiding practises were more frequently discussed during pregnancy than health-promoting practises, by younger and older mothers. Health-promoting practises and risk-avoiding practises were equally likely to be discussed postpartum by younger and older mothers (table 1).

### (d) Relationship of traditional medical practises to biomedical recommendations

During pregnancy and postpartum, the vast majority of health practises were neutral with respect to specific biomedical recommendations, some were consistent and some were inconsistent with biomedical recommendations (table 1). Examples of practises that are consistent with biomedical recommendations include avoiding hard labour during pregnancy, side sleeping while pregnant, and immediate skin-to-skin contact and initiation of breastfeeding. Examples of practises that are inconsistent with biomedical recommendations include bathing the mother and child within 24 h of birth, applying mustard oil to the umbilical cord stump, and colostrum taboos (e.g. discarding instead of feeding colostrum to the newborn). A relatively larger portion of traditional health practises are inconsistent with biomedical recommendations postpartum than prepartum because there are independent risks to mother and infant postpartum and infants are highly susceptible to infection (e.g. umbilical cord stump infection owing to contact with water or traditional ointments) and food or water transmitted infections (e.g. feeding newborns unsterilized water or cows' milk). Examples of practises that are neutral with respect to biomedical recommendations include pregnant women applying warm water compresses with carom seed to their bodies, placing an iron object by the doorway of the mother's room to protect from evil eye, tying a string around the infant's wrist for supernatural protection and tonsuring (head-shaving) to rid the infant of impurities.

### (e) Coexistence of traditional and biomedical health practises

There are multiple examples of replacement, conflict and complementarity of traditional and biomedical health practises. An example of replacement in the context of umbilical cord care is mothers who reported no longer applying mustard oil to the infant's umbilical cord stump following birth (traditional perinatal health practise), in favour of applying nothing after the wound has dried, which is consistent with the biomedical recommendation for umbilical cord care. An example of conflict is mothers who report continuing to apply mustard oil on the infant's umbilical cord stump following birth, which is inconsistent with the biomedical recommendation for umbilical cord care. An example of complementarity is mothers who report applying mustard oil on the rest of the infant's body, but avoiding the umbilical cord stump, which incorporates traditional perinatal ritual massage of the infant following birth, *and* is consistent with the biomedical recommendation for umbilical cord care.

An example of replacement in the context of breastfeeding is mothers who reported no longer waiting for the Hindu *pandit* or Muslim *maulana* to initiate immediate breastfeeding (traditional perinatal ritual), which is consistent with the biomedical recommendation for immediate and exclusive breastfeeding. An example of conflict is mothers who reported delaying breastfeeding until a Hindu *pandit* or Muslim *maulana* visits the mother and infant at home (traditional perinatal ritual), and instead feeding the newborn a mixture of cows' milk and water, inconsistent with the biomedical recommendation for immediate and exclusive breastfeeding. An example of complementarity is mothers who reported calling a Hindu *pandit* or Muslim *maulana* on a mobile phone to receive blessings from the hospital immediately after birth, which incorporates traditional perinatal ritual religious blessings to initiate breastfeeding, *and* is consistent with biomedical recommendation for immediate and exclusive breastfeeding.

## 4. General discussion

Our objective was to examine the local cultural ecology of health associated with mitigating risk during the perinatal period in Bihar, a population experiencing high levels of risk to mothers and infants. We documented a substantial number of health-related concerns during the pregnancy and postpartum periods and the physical and supernatural threats associated with them. We also described traditional and biomedical practises believed to mitigate risk and promote health for mother and infant.

Our data demonstrated that the perinatal period, like all fitness-relevant events, is marked by an extensive number of traditional health practises. The large majority of practises in our dataset have the goal of avoiding risk, which is consistent with the proposal that traditional health practises such as perinatal rituals function as a response to perceived threat. The extent to which health-related practises were explained as risk avoiding versus health promoting varied between different points in the perinatal period. For example, health practises during pregnancy were primarily explained in terms of risk avoidance. By contrast, explanations for health practises after birth were evenly distributed between risk avoidance and health promotion.

Mothers reported an extensive number of traditional medical practises with the goal of reducing physical and supernatural risks to mother and child. Practises associated with placing visible markers or objects on the body were commonly associated with avoiding the ‘gaze' of evil eye. Common taboos observed and superstitions practised to reduce risk of miscarriage include avoiding crossing paths with the jiloi bird and applying kohl around the eyes of the infant. Mothers also mentioned taking precautions to avoid elevated risks during solar eclipses, which are associated with supernatural threat. During a solar eclipse, a thread equivalent to the height of the pregnant woman should be hung on the wall. In the postpartum period, horoscope rituals are practised to avoid the threat of malefic planets. Multiple mothers discussed a perinatal ritual practised if a child is born in 27th nakshatra. The father sees the child in the shadow of oil or ghee, and the infant is bathed with water from 27 wells. Pots are made from 27 bamboo trees to worship the god. Seven types of fruits and sweets are prepared. Mothers also mentioned taking precautions during periods of impurity. New mothers and infants are considered impure and thus sleep separately from the family after birth to avoid contaminating others.

The extensive use of traditional medical practises for protection from supernatural harm in Bihar is consistent with previous research in South Africa and Haiti on the use of traditional medical practises to protect people from witchcraft [7,16]. Many of the perinatal rituals documented in the study, including initiation rituals (e.g. Chhathi) and horoscope rituals also included practises believed to avoid supernatural threat (e.g. applying kohl around the eyes and avoiding images of goddesses). This suggests that the emphasis on risk mitigation in traditional medicine is common across highly diverse cultural ecologies of health.

Many of the perinatal rituals documented in this study, such as Chhathi, involved repetition and high levels of procedural detail, consistent with previous research on perceptions of ritual efficacy. Supernatural agents were also frequently associated with both Hindu and Muslim perinatal ritual practises, also consistent with previous research associating supernatural agency with perceptions of ritual efficacy [6,39]. The involvement of the family and local religious leaders, the special clothing and artefacts worn by mother and child, and the consistency in the practises associated with Chhathi ceremony across Hindu and Muslim mothers provide convergent support for the social functions of initiation rituals.

Traditional medical practises based on associational magic [45] were also reported. For example, to reduce the risk of a child being bitten by a scorpion, a mother mentioned that a scorpion is pasted on the wall with cow dung and after delivery the dung cake is burnt on fire. Another mother mentioned that during an eclipse, a pregnant woman should not cut hair or rope to avoid her infant being born with physical deformities associated with cuts, such as a cleft lip. Burying the umbilical cord stump to ensure the longevity of family ancestry was a frequently mentioned traditional medical practise.

We documented a number of ways in which traditional and biomedical health practises compete, conflict and coexist. There were infrequent examples of conflict between traditional and biomedical practises, such as women who gave birth in facilities applying mustard oil to the cord stump during ritual massage of the infant or delaying the initiation of breastfeeding for several days for the *pandit* or *maulana* to bless the infant. Most traditional medical practises were neutral or unrelated, and often coexisted alongside biomedical practises. For example, many mothers who gave birth in hospitals, a recommended biomedical practise, also used traditional superstitions to ward off the threat of evil eye, such as bringing iron objects to delivery and placing them in the doorway of the home.

We propose that the relationships between traditional and biomedical practises in the context of cultural ecologies of health is critical to improving the efficacy of health education interventions and policies. Practises associated with traditional health are widely assumed to negatively impact health, often without evidence. We show that the vast majority of traditional perinatal health practises are consistent or neutral re biomedical health practise. Future research should examine how the social capital associated with traditional medicine can be harnessed to improve the efficacy of biomedical health interventions. For example, many of the recommended biomedical health practises, such as immediate breastfeeding, institutional delivery and cord care can be accommodated within existing perinatal rituals. For example, using the important social functions of birth rituals like Chhathi provides a unique opportunity for local health activists, educators and practitioners to communicate with new mothers and families about health-promoting behaviours. Local health activists and educators often attend Chhathi ceremonies and could use these opportunities to provide information about recommended biomedical practises to mothers and families. Recognizing the similarities between traditional medicine and biomedicine has the potential to provide unique insight into how both healthcare systems function within local ecologies of health. Both operate as ritualistic remedies; they are socially transmitted, causally opaque, and used to mitigate risk.

Examining the extent to which traditional perinatal medical practises are consistent, inconsistent or unrelated to biomedical health practises and documenting the extent to which traditional and biomedical health practises compete, conflict and coexist, provides unique insight into cultural ecologies of health. Our dataset provides evidence for the relationships between risk and health practises during the perinatal period and reveals strong associations between health practises and avoiding negative outcomes. We argue that a comprehensive understanding of traditional health practises can improve the efficacy of health interventions and encourage future research on maternal and child health to examine traditional medicine worldwide.

## Data accessibility

Data reported in this paper are available in the following Open Science Framework (OSF) project repository: https://osf.io/mtcp9/. The raw data and our coding is publicly available at this OSF site. We did not post code because the analysis reported in this paper consists of counts and percentages.
